# Mechanical stretching induces fibroblasts apoptosis through activating Piezo1 and then destroying actin cytoskeleton

**DOI:** 10.7150/ijms.81666

**Published:** 2023-04-24

**Authors:** Yang Li, Lu Li, Bingshu Li, Wenxin Liao, Tingting Liu, Fujin Shen, Li Hong

**Affiliations:** 1 Department of Gynecology and Obstetrics, Renmin Hospital of Wuhan University, Wuhan 430060, Hubei Province, P. R. China; 2 Department of Gynecology and Obstetrics, the People's Hospital of Three Gorges University/ the First People's Hospital of Yichang

**Keywords:** Stress urinary incontinence, Mechanical stretching, Actin cytoskeleton, Apoptosis

## Abstract

The anatomical positions of pelvic floor organs are maintained by ligaments and muscles. Stress urinary incontinence (SUI) occurs when the pelvic floor tissues are repeatedly stimulated by excessive mechanical tension that exceeds the bearing capacity of ligaments or muscles. Besides, cells respond mechanically to mechanical stimulation by reconstituting the Piezo1 and cytoskeletal system. The aim of this study is to determine how Piezo1 and actin cytoskeleton are involved in the mechanized stretch (MS) induced apoptosis of human anterior vaginal wall fibroblasts (hAVWFs) and the mechanism. A four-point bending device was used to provide mechanical stretching to establish a cellular mechanical damage model. The apoptosis of hAVWFs cells in non-SUI patients was significantly increased by MS, which exhibited apoptosis rates comparable to those of SUI patients. Based on these findings, Piezo1 connects the actin cytoskeleton to the apoptosis of hAVWFs cells, providing an idea for the clinical diagnosis and treatment of SUI. However, the disassembly of the actin cytoskeleton suppressed the protective effect of Piezo1 silencing on MS. Based on these findings, Piezo1 connects the actin cytoskeleton to apoptosis of hAVWFs, providing new insight for the clinical diagnosis and treatment of SUI.

## Introduction

International Continence Society (ICS) defines stress urinary incontinence (SUI) as the involuntary spilling of urine during a sudden increase in abdominal pressure, such as exercise, sneezing or coughing. Epidemiological data in China showed that the total prevalence rate of SUI was 18.9%, and the incidence increased with age, mainly occurring in young, middle-aged and elderly women during the puerperal period 1. Especially in the context of the global population aging, the incidence of SUI is expected to increase significantly. However, the specific pathogenesis of SUI remains unclear, leading to a lot of confusion and controversy in clinical diagnosis, treatment, and prevention. Therefore, the study of its pathogenesis has important clinical and social significance at this point. The current mainstream view is that the relaxation of pelvic floor supporting structure can lead to SUI. In addition to the degenerative lesions of the pelvic floor supporting structure caused by aging, pregnancy, childbirth, constipation and obesity can all lead to the increase of pelvic and abdominal pressure, resulting in the abnormal increase of pelvic tissue mechanical force, which will cause mechanical damage to the pelvic support structure and ultimately lead to the occurrence of SUI 2, 3.

The behavior of cells can be influenced by a variety of external mechanical signals, including stretching, shear stress, matrix stiffness, osmotic stress, and the topology of the substrate. They can integrate mechanical signals with genetic and chemical information 4. In macroscopic terms, the growth, dynamics, and homeostasis of organisms are governed by the forces of interaction between cells and tissues. As a result, biological processes are heavily influenced by mechanical forces, but we know little about how they do so. In cells, mechanical forces are detected by specialized molecules, which transmit biochemical signals to form molecules and genetic processes. Piezo1 has been shown to respond to a wide range of mechanical cues, including membrane stretch, cell indentation, and substrate stiffness 5, 6. The Piezo family consists of two family members, Piezo1 and Piezo2, which perform functions in different tissues and organs. Piezo1 is mainly expressed in unstimulated cells 7, playing an important role in the conversion of mechanical signals on the plasma membrane 8. In addition, there is a close relationship between piezo1 and cytoskeleton, and they interact with each other to cope with mechanical forces 9, 10.

Both human and animal studies show an increased apoptosis in SUI subjects compared to controls 11-13. Our previous experiments also showed that the invasive mechanical stress could significantly decreased the number of fibroblasts 13, 14, indicating that apoptosis of pelvic floor fibroblasts was an important trigger of SUI. In addition, a large of studies have demonstrated that the onset of SUI is closely related to the remodeling of extracellular matrix (ECM) 15, 16, and the secretion of major components of ECM, such as collagen and cytokines, are strongly associated with the pelvic floor fibroblasts 17. Although numerous studies have strongly suggested that mechanical stretching induced apoptosis, the underlying mechanism of mechanical stretching leading to SUI remained unclear. In this study, we found that excessive mechanical stretching destroyed the actin cytoskeleton and induced the apoptosis of human anterior vaginal wall fibroblasts (hAVWFs) through the activation of Piezo1. Through this study, we hope to provide a theoretical basis for finding new strategies to prevent or treat SUI.

## Materials and Methods

### Patients and sample collection

The present study was approved by the Ethics Committee of Renmin Hospital of Wuhan University (Wuhan, China), and verbal and written informed consent was obtained from all patients prior to participation. A total of 23 patients who underwent gynecological surgery in our hospital were selected from March 2020 to December 2021, which including 10 patients with SUI (with anterior vaginal wall prolapse or cystocele, POP-Q Ⅲ ~ Ⅳ: Ba > + 1, as SUI group), and the other 13 patients underwent total hysterectomy for benign tumors (control group). During the operation, the tissue specimens (0.5*0.5*0.5cm^3^) were obtained from the anterior vaginal wall, and then sent to the laboratory within half an hour for follow-up experiments. There was no evidence of connective tissue disease, endometriosis, or gynecologic malignancy in all patients recruited. We excluded patients who had undergone surgery on the anterior vaginal wall site or applied estrogen in the past three months.

### Primary cell culture

We cultured and purified hAVWFs according to our previous description 18. Briefly, the anterior vaginal wall tissues were cut into pieces (approximately 1 mm^3^), placed in culture bottles and digested with Type I Collagenase (ThermoFisher, catalog No. 17100-017) and trypsinase (Servicebio, catalog No. G4001). The fibroblasts were grown in serum-free DMEM (HyClone, catalog No. SH30243) supplemented with 20% fetal bovine serum (HyClone, catalog No. SV30087), and 100 U/ml penicillin/streptomycin (Beyotime, catalog No. ST488) at 37 °C in an incubator with 5% CO2. After passage at 85% confluency, hAVMFs were used for subsequent experiments at passages 3 to 6. For the latrunculin A (Lat-A, a potent inhibitor of F-actin assembly; Cayman, catalog No. 10010630) experiment, hAVMFs were treated with Lat-A (0.5 μM) for 24 h.

### Immunohistochemistry (IHC)

The paraffin-embedded human anterior vaginal wall samples were cut into 4μm-thick sections, which were then dewaxed with xylene and rehydrated. Incubation with anti-Piezo1 (Affinit, 1:200, catalog No. DF12083) overnight at 4°C followed by secondary antibody (Maxim Biotechnologies, Fuzhou, China) incubation for 1 hour at room temperature was carried out after antigen repair using microwave or citrate antigen retrieval solutions. The DAB kit (Maxim Biotechnologies, Fuzhou, China) was used as the chromogenic agent, and hematoxylin was used for nuclei counterstaining. Dehydration and sealing were performed in sequence, and the positive expression quantification was performed using ImageJ 1.48r software.

### Mechanical stretching application

**Figure 1:**
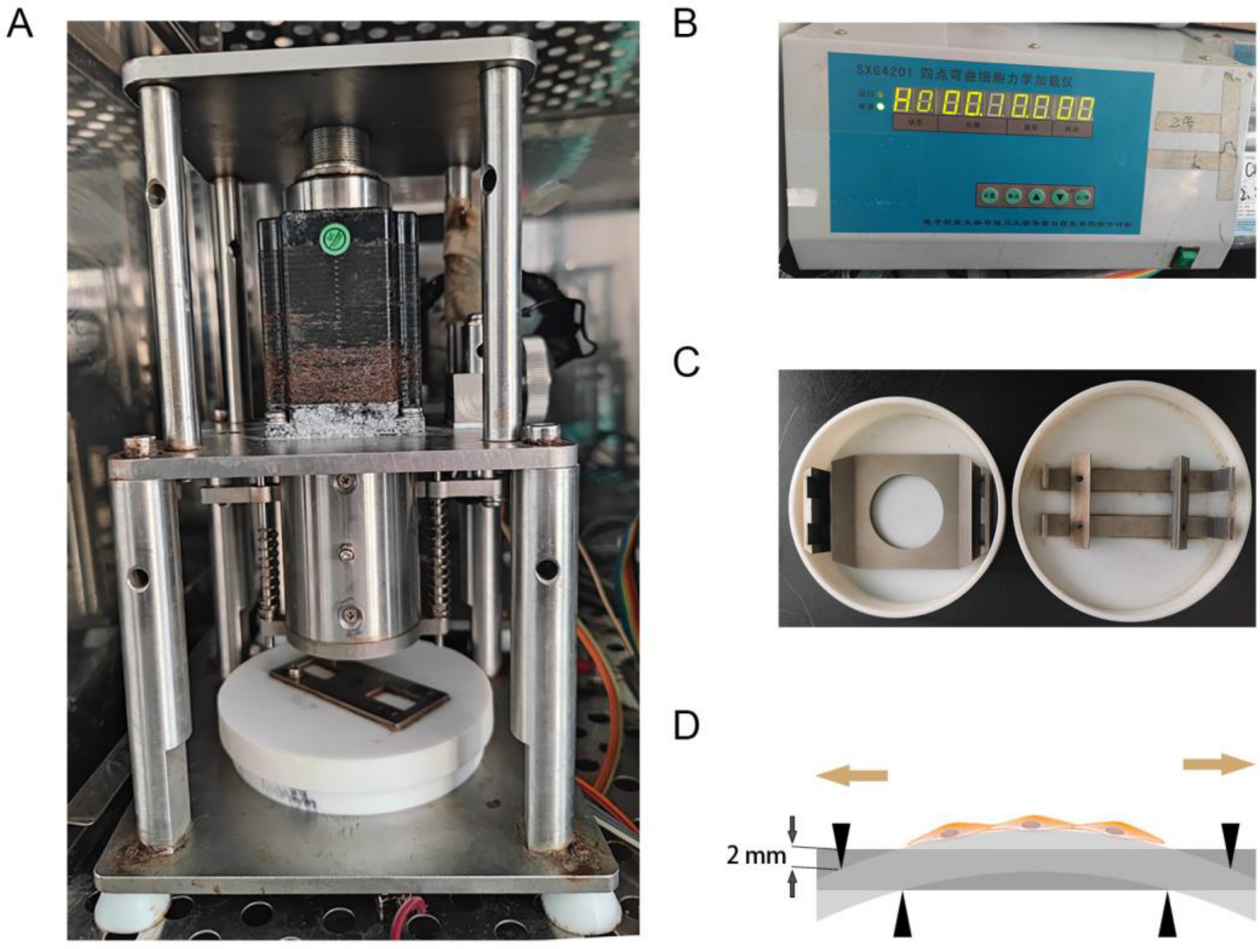
** Mechanical stretching application. A**, Mechanical dynamic system; **B**, Drive control system; **C**, Mechanical stretching-loaded petri dish; **D**, schematic diagram of mechanical stretching loading on hAVMFs.

As previously described 14, the mechanical stretching loaded on hAVMFs was generated by a four‑point bending device (SXG4201; Chengdu Miracle Chemicals Co., Ltd., Chengdu, China). Basically, the device is consisited of three parts: mechanical dynamic system, drive control system, mechanical stretching-loaded petri dish (Figure 1 A, B, C). The parameters of mechanical force were set to 2666 με (the loading displacement was 2 mm) as the magnitude of the force on the sample at a frequency of 0.1 Hz for 4 h. (Figure 1 D).

### Phalloidin staining

Actin polymerization mainly forms F-actin, and the depolymerization state is monomer G-actin. So we were able to measure the amount of F-actin in the aggregate state indirectly reactive depolymerization state by Phalloidin staining. After different treatments, Fluor-phalloidin (CST, catalog no. 8953) was incubated at room temperature for 1 h after hAVWFs had been fixed with 4% fresh paraformaldehyde for 15 minutes and permeabilized with 0.4% Triton X-100 for 5 minutes. The nuclei were stained with DAPI and observed under fluorescence microscope. Quantitative analyses of indicated stains were performed by ImageJ software.

### Flow cytometry

The apoptosis of hAVWFs in each group was detected by flow cytometry. After digestion with pancreatin and washing with PBS for three times, the hAVWFs were resuspended with 100L binding buffer. After that, for each group, 5 μL of PE Annexin V and 7μL of 7-AAD were added in accordance with the instructions on the PE Annexin V Apoptosis Detection Kit I (BD Biosciences, catalog No. 559763, USA). Apoptosis was detected by flow cytometry after gently resuspending the hAVWFs and incubating them for 15 minutes at room temperature in the dark.

### Small interfering RNA

According to the manufacturer's instructions, a transfection agent (GeneChem Co., Ltd, Shanghai, China) was used to transient transfect primary hAVWFs with Piezo1-targeting siRNA. In order to assess knockdown efficiency, Western blots were used to detect changes in the expression of each protein.

### Immunofluorescence

The morphology of hAVWFs was characterized as spindle-shaped and they were identified by Immunocytochemistry staining which showed a positive staining for vimentin and negative staining for cytokeratin. Cells from passages 3-6 of hAVWFs were cultured to 50% confluent on ventricular slides. As mentioned before^11^, after fixation with 4% paraformaldehyde and cell membrane permeabilization with Triton X100, hAVWFs were incubated overnight with anti-Piezo1 (Affinit, 1:200, catalog No. DF12083), anti-vimentin (Santa, catalog No. sc-6260; 200 μg/ml) and anti-cytokeratin (Santa, catalog No. sc-376224, 200 μg/ml) at 4 °C. Nuclei were stained with DAPI and observed under a fluorescence microscope. Quantitative analyses of indicated stains were performed using ImageJ software.

### TUNEL assay

The paraffin sections of the anterior vaginal wall tissue samples were baked in an oven at 60°C for 1 hour and the wax was removed with water, xylene and different concentrations of ethanol (100%/90%/80%70%). Then paraffin sections were stained using an *in situ* TUNEL assay kit (Roche, Basel, Switzerland) according to the manufacturer's instructions to identify apoptotic cells. The nuclei were stained with DAPI. TUNEL-positive nuclei (red) and total cells (blue) were imaged using a fluorescence microscopy and analyzed by ImageJ 1.48r software (NIH, Bethesda, MD, USA) in at least five randomly selected fields per section (100× magnification). The apoptotic index is expressed as the ratio of apoptotic cells to total hAVWFs of DAPI-stained nuclei.

### Western blotting

After exposure to different treatments, proteinase inhibitors were added to RIPA lysis buffer to separate the total proteins of hAVWFs. Samples were used to evaluate protein quantification with a BCA assay kit (Beyotime Institute of Biotechnology, catalog No. P0010, Haimen, China). In total, 20 µg of total protein were separated by SDS-PAGE then transferred to a polyvinylidene fluoride membrane (PVDF, Merck KGaA) with 10% SDS-PAGE, then blocked with 5% skim milk-TBST (TBS with 0.1% Tween 20). The rabbit primary antibody: anti-Piezo1(1:1000, catalog No. DF12083) and anti-GapDH (1:2000, catalog No. ab9485, as an internal reference control), purchased from Affinity Biosciences and Abcam, were kept overnight at 4˚C. Then, the membrane was incubated with relevant HRP-conjugated secondary antibody for 1 h at room temperature. The reactive bands were exposed using 500 μL Western Lightning Plus-ECL (Perkin Elmer; catalog No. NEL105001EA) on a ChemiDoc MP Imaging System (Bio-Rad). The strip density of each sample was normalized according to the density of GAPDH, and data were obtained from three experiments.

### Quantitative RT-PCR

After different treatments, RNA was isolated from hAVWFs using TRIzol® reagent (Invitrogen; Thermo Fisher Scientific, Inc., Waltham, MA, USA) per the manufacturer's instructions. After the detection of RNA concentration, 100 ng of RNA was used to transcribe cDNA with a Revert Aid First Strand cDNA Synthesis kit (catalog No. k1622; Thermo Fisher Scientific, Inc. USA) per the manufacturer's instructions. Beijing SBS Genetech Co., Ltd. (Beijing, China) provided the primers for amplification. Quantitative RT-PCR was performed on an Applied Biosystems 7500 Real-Time system (Applied Biosystems, Thermo Fisher Scientific, Inc. USA) using SYBR® Premix Ex Taq™ reagent (catalog No. DRR041; TakaRa Bio, Inc., Otsu, Japan) per the manufacturer's instructions. The mRNA expression levels of target genes were quantified by comparing them to GAPDH expression levels. All RT-PCR reactions were performed in triplicate using biological triplicate technology to ensure accuracy.

### Statistical analysis

The GraphPad Prism 6 (GraphPad Prism INC, CA, USA) program was used to assess significant differences between groups using one-way analysis of variance (over two groups), followed by a Tukey's posttest to make multiple comparisons within the test group. The differences were considered significant with P < 0.05.

## Results

### Identification of hAVWFs

**Figure 2:**
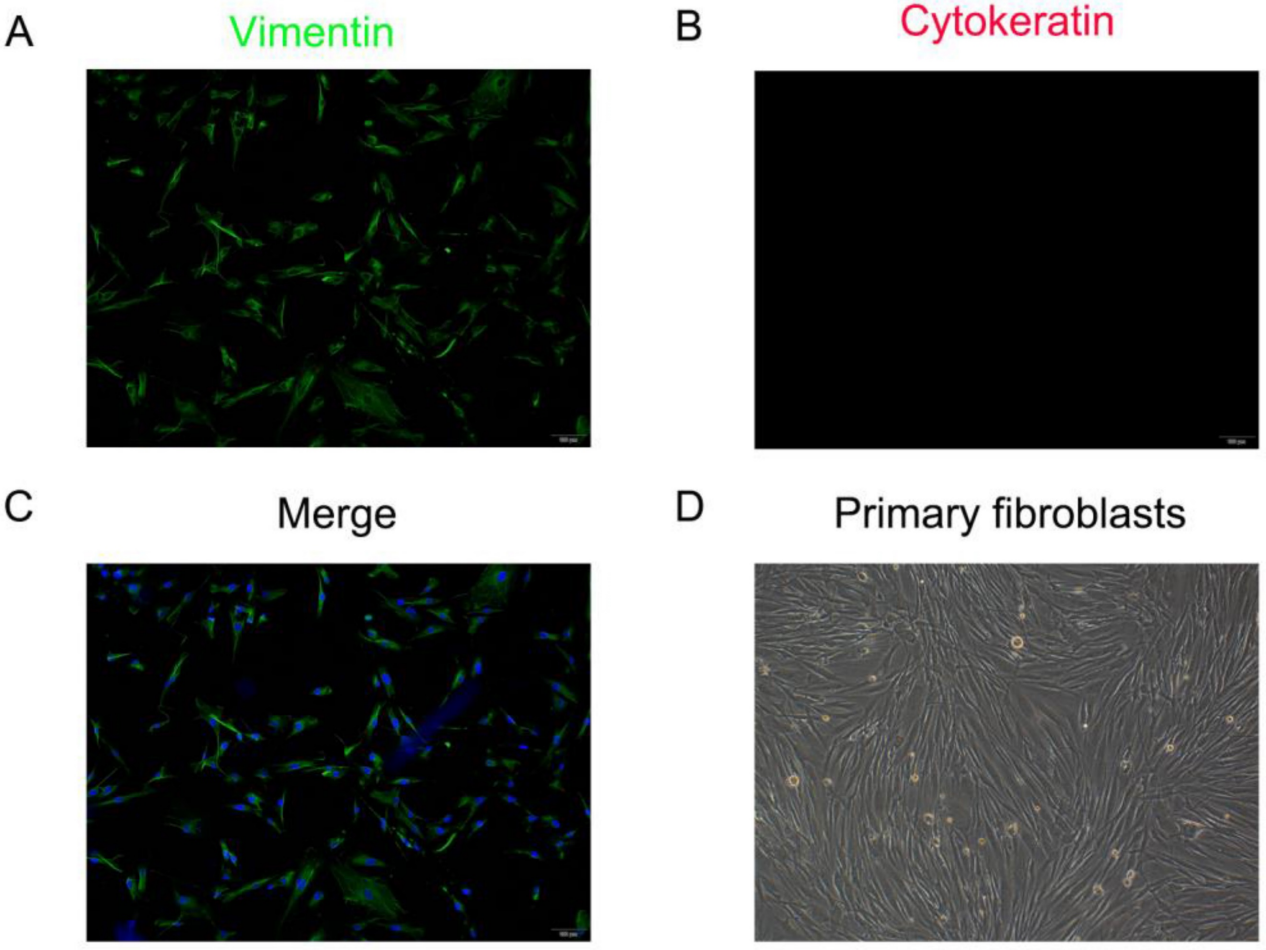
** Identification of primary human anterior vaginal wall fibroblasts (hAVWFs). Immunofluorescence staining for vimentin (A)**, cytokeratin **(B)** in cultured hAVWFs and the merged image with vimentin, cytokeratin and DAPI staining **(C)**. **(D)**, Primary cultured hAVWFs was observed by light microscopy. (magnification: 100×)

The primary fibroblasts were isolated from the anterior vaginal wall which showed vimentin is strongly expressed in the cytoplasm, while cytokeratin is not stained under immunofluorescence staining. Cells exhibited spindle- or long-spindle-shaped and some were rod-like under an inverted microscope (Figure 2). Under the inverted microscope, the cells were spindle or long spindle, and some were rod-shaped.

### The apoptosis rate of anterior vaginal wall samples increased in SUI patients

**Figure 3:**
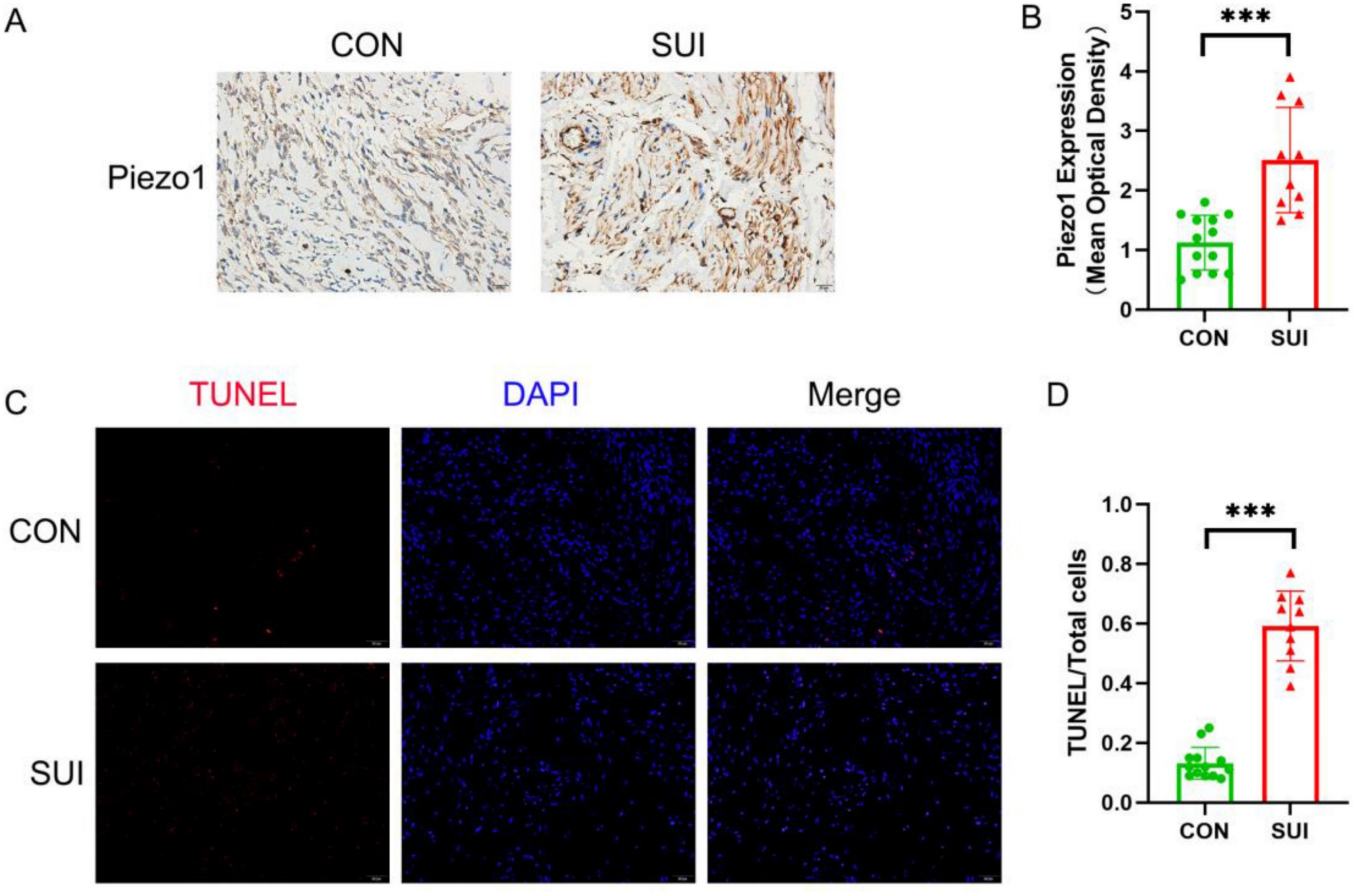
** Detection of Piezo1 expression and apoptosis rate of the anterior vaginal wall tissue. A**, Piezo1 immunohistochemistry staining of the anterior vaginal wall tissue, magnification: ×200; **B**, semiquantitative assay of Piezo1 expression using Image J; **C**, TUNEL staining of the anterior vaginal wall tissue, magnification: 200×; **D**, The ratio of TUNEL-positive cells to the total number of cells. *** indicates p < 0.001. (CON: the anterior vaginal wall tissue obtained from patients without SUI; SUI: the anterior vaginal wall tissue obtained from patients with SUI).

We observed that the level of Piezo1 in the anterior vaginal wall of SUI patients was higher than that in the control group (Figure 3 A, B). It is noteworthy that the apoptosis rate of SUI group is significantly higher than that of the control group, and has statistical significance (Figure 3 C, D). According to the above data, Piezo1 may be involved in the increased apoptosis of anterior vaginal wall cells in patients with SUI.

### Mechanical stretching induced the disintegration of actin cytoskeleton and increased the apoptosis of hAVWFs

**Figure 4:**
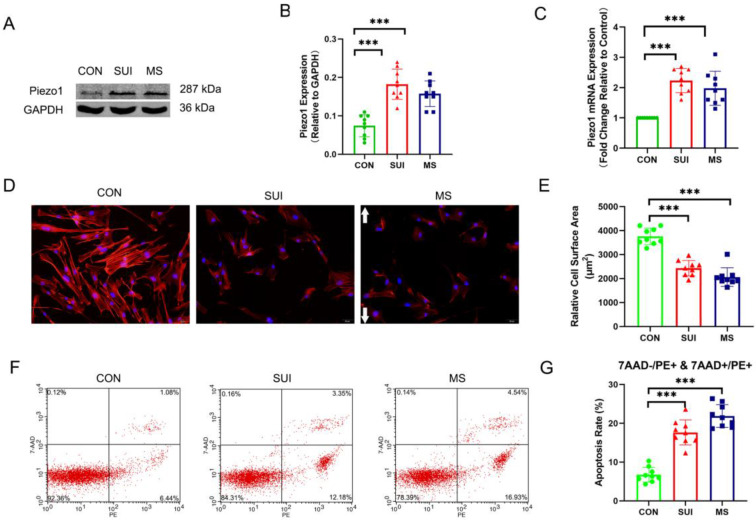
** The effects of mechanical stretching on apoptosis rate and actin cytoskeleton disassembly in hAVWFs. A**, Piezo1 expression in hAVWFs was detected by Western blotting and normalized into GAPDH; **B**, Quantity One was used to quantify the energy band strength; **C**, Piezo1 mRNA levels in hAVWFs was quantified by real-time RT-PCR and normalized to GAPDH; **D**, hAVWFs were stained with phalloidin and imaged by fluorescence microscopy. Red fluorescence delineates the cell cytoplasm; blue fluorescence delineates nuclei (magnification: 200×); **E**, The relative cell surface areas were quantified by ImageJ software; **F**, Cell apoptosis was assessed by flow cytometry analysis; **G**, Quantified apoptosis rates in each group. *** indicates p < 0.001; white arrows show the direction of mechanical stretching. (CON: hAVWFs isolated from patients without SUI; SUI: hAVWFs isolated from patients with SUI; MS: hAVWFs isolated from patients without SUI that were exposed to mechanical stretching).

In order to investigate the effects of abnormal mechanical forces on fibroblasts, we developed a cellular mechanical stretching loading model. As expected, we discovered that the levels of Piezo1 was significantly increased (Figure 4 A, B, C). An actin filament is formed by reversibly polymerizing 42 kDa globular proteins (G-actin) into filaments (F-actin, which can combine with phalloidin). MS suppressed the expression of F-actin, which is an indirect sign of actin cytoskeletal depolymerization. (Figure 4 D, E). Apoptosis rates were calculated using PE (a fluorescein used to label Annexin-V for early and late apoptotic cells) positive cells as markers. 7-AAD was negative in early apoptotic cells and positive in late apoptotic cells. Apoptosis induced by MS was significantly increased (Figure 4 F, G). Notably, the hAVWFs of SUI patients showed similar differences in Piezo1 expression, apoptosis rate and actin cytoskeletal decomposition between the hAVWFs of the MS control group (Figure 4 A, B, C, D, E, F, G). Accordingly, the mechanical force may be involved in the occurrence and development of SUI, leading to the apoptosis and decomposition of the actin cytoskeleton in hAVWFs.

### Piezo1 Silencing alleviated MS-induced apoptosis

**Figure 5:**
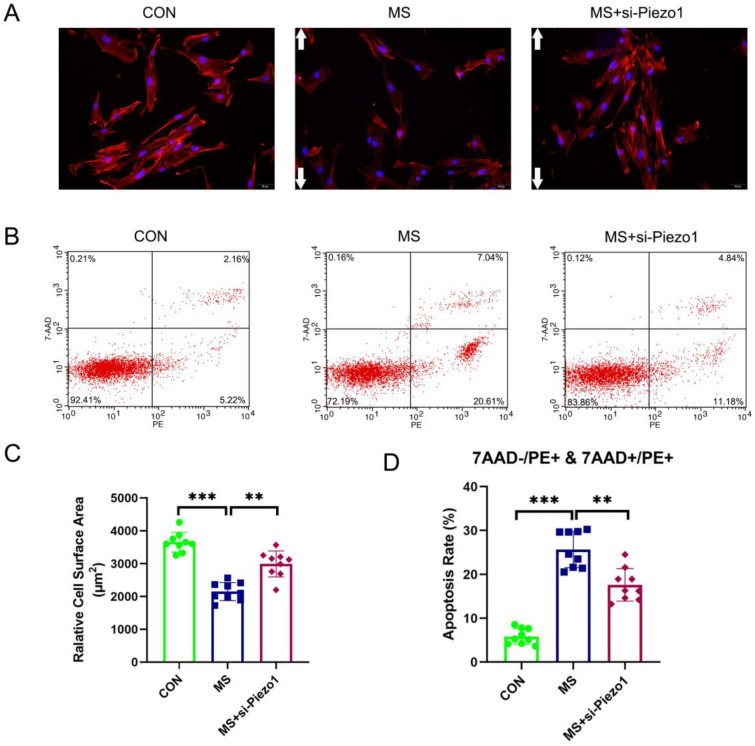
** The effects of mechanical stretching on apoptosis and actin cytoskeleton disassembly after Piezo1 silencing. A**, hAVWFs were stained with phalloidin and imaged by fluorescence microscopy (magnification: 200×); **B**, Cell apoptosis was detected by flow cytometry analysis; **C**, Relative cell surface areas were quantified by ImageJ software; **D**, Quantified apoptosis rates in each group. ** indicates p < 0.01 and *** indicates p < 0.001; white arrows show the direction of mechanical stretching. (CON: hAVWFs isolated from patients without SUI; MS: hAVWFs isolated from patients without SUI and exposed to mechanical stretching; MS+si-Piezo1: hAVWFs isolated from non-SUI patients with Piezo1 silencing and exposed to mechanical stretching).

The aim of this study was to demonstrate the importance of Piezo1 in MS-induced apoptosis by evaluating the functional effects of Piezo1 silencing. The results showed that MS insult markedly increased the apoptosis rate and actin cytoskeleton disassembly of hAVWFs compared with the control group. However, the knockdown of Piezo1 significantly reversed the increase induced by MS (Figure 5 A, B, C, D). The silencing of Piezo1 seemed to provide a protection against MS-induced injury cell damage, suggesting that Piezo1 may play an important role in MS-induced apoptosis and actin cytoskeleton disassembly.

### Disassembly of actin cytoskeleton suppressed the protective effect of Piezo1 silencing from MS

**Figure 6:**
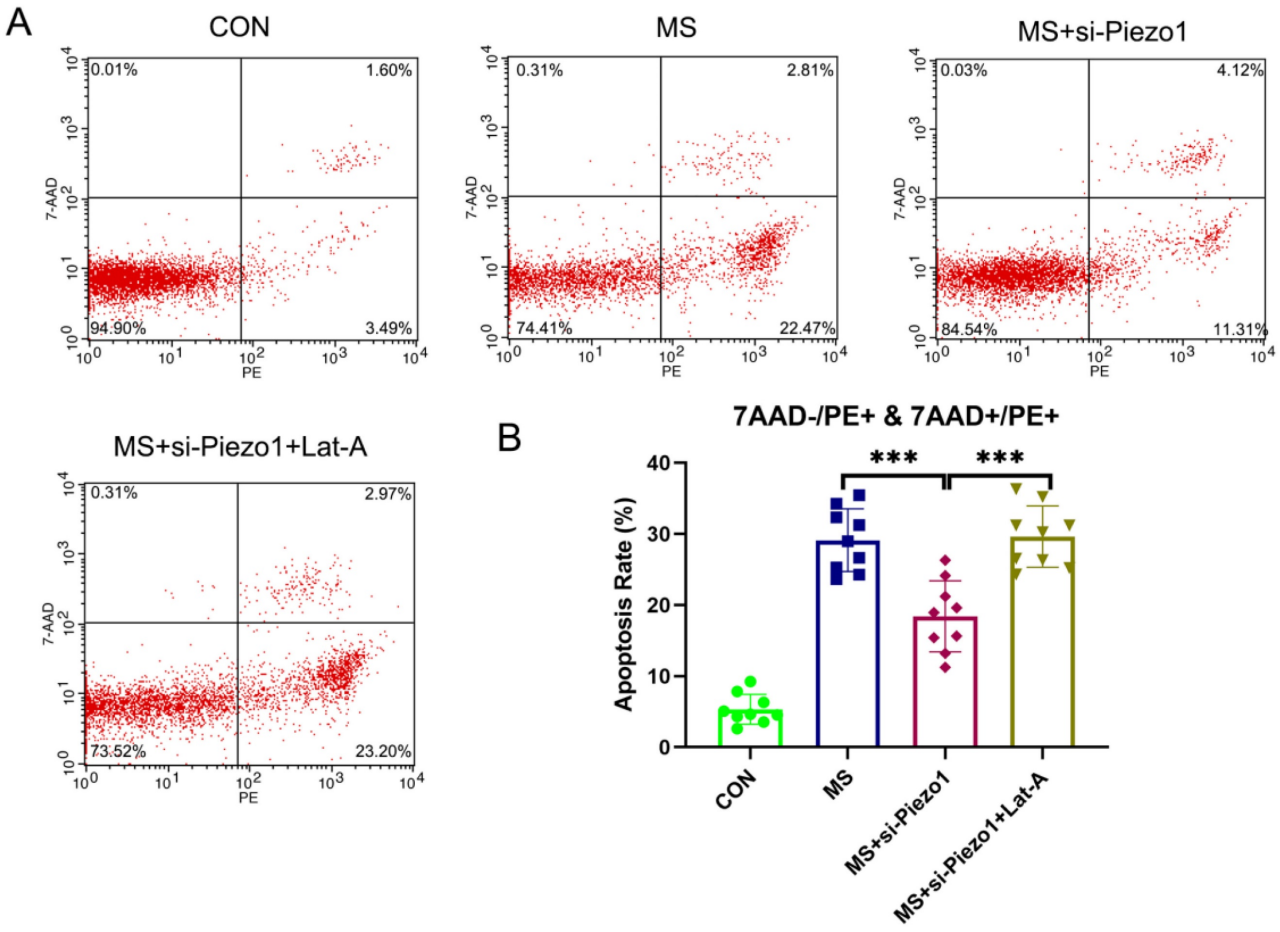
The disassembly of the actin cytoskeleton suppressed the protective effect of Piezo1 silencing from MS. **A**, Cell apoptosis was detected by flow cytometry analysis; **B**, Quantified apoptosis rates in each group. *** indicates p < 0.001. (CON: hAVWFs isolated from patients without SUI; MS: hAVWFs isolated from patients without SUI and exposed to mechanical stretching; MS+si-Piezo1: hAVWFs isolated from non-SUI patients with Piezo1 silencing and exposed to mechanical stretching; hAVWFs isolated from non-SUI patients with Piezo1 silencing and exposed to mechanical stretching and Lat-A).

So, under mechanical stretching, does Piezo1 induce apoptosis by increasing the disassembly of actin cytoskeleton? To solve this problem, the actin cytoskeleton was disrupted using Lat-A, which sequesters monomeric actin and causes actin microfibrils to depolymerize rapidly. The silencing of Piezo1 seemed to provide a protection against MS-induced damage. However, the disassembly of actin cytoskeleton suppressed the protective effect of Piezo1 silencing on MS (Figure 6 A, B). Based on the above data, it is evident that the overexpression of Piezo1 contributed to the depolymerization of actin cytoskeleton to participate in MS-induced apoptosis.

## Discussion

The molecular mechanism of SUI has not been fully elucidated so far. From the perspective of anatomy, the key mechanism leading to SUI is the weakening of urethral support function and the defect of urethral inherent sphincter function. Muscles, ligaments and fascia of the pelvic floor work together to provide support for the urethra. Fascia and ligaments achieve urinary self-incontinence by maintaining the position of the bladder neck and urethra and the closure of the urethra. The pelvic floor tissue of women is subject to complex biomechanical changes caused by changes in abdominal pressure during pregnancy and delivery. Maintaining the normal structure and function of the pelvic floor depends on the balance of forces produced by the pelvic floor muscles and ligaments. The "whole theory" and "hammock hypothesis" also emphasize that the pelvic floor fascia ligament plays an important role in resisting external forces. Studies on the pathogenesis of SUI suggest that the key factors are closely related to the relaxation of supporting structures, such as the pelvic floor fascia ligaments 19, which are mainly formed largely by fibroblasts. Kokcu et al found that the number of fibroblasts in patients with pelvic floor relaxation was significantly lower than that in patients with non-prolapse 20. Traumatic mechanical force can significantly inhibit the proliferation of fibroblasts 11. In conclusion, the decrease in the number of pelvic floor fibroblasts is an important cause of SUI. In this study, the apoptosis rate of anterior vaginal wall fibroblasts was significantly increased in both SUI and non-SUI patients after mechanical stretching. These findings suggest that the death of fibroblasts in pelvic floor supporting tissue caused by excessive mechanical stimulation may be an important cause of SUI, but its specific mechanism still needs to be explored.

The dynamic mechanical behavior of living cells is based on the Laws of Newtonian mechanics. Fibroblasts have mechanosensitive characteristics and are able to accept and adapt mechanical forces in response to exposure to mechanical stimuli because they convert mechanical forces into chemical signals, as they transduce a mechanical force into chemical signals 4. The mechanical force exerted on cells is sensed by specialized molecules. However, there is still a gap in the understanding of the specific molecular mechanism in this process. Over the past decade, scientists have carried out a large number of *in vivo* or *in vitro* studies to expound how cells sense mechanical forces and translate them into chemical-signaling events. The discovery of Piezo is one of the important achievements in this field. Piezo is an evolutionarily conserved molecule involved in many signal transduction pathways, such as cell development, migration and differentiation, which is activated in many different cell types and in response to a diverse array of cellular forces 21, 22. Piezo1 is mainly expressed in non-excitable cells, which plays an important role in translating mechanical forces into chemical signaling 7, 8. In terms of molecular structure, Piezo1 trimeric forms a remarkable three-bladed, propeller-like architecture, exhibited exceptional mechanical behaviour that originate from their unique molecular topologies 23. In articular chondrocytes, excessive mechanical tension activated Piezo1, and then upregulated caspase-12 further activating the caspase cascade and leading to apoptosis 24. Meanwhile, it has also been reported that GsMTx4 can inhibit Piezo1 activity in the urothelium, affecting the arterial remodeling on which Piezo1 depends and reducing cell death caused by high-strain mechanical damage 25. In the present study, we found that the expression of Piezo1 and apoptosis rate were significantly increased in the SUI group when compared to healthy controls. Moreover, the expression of Piezo1 and apoptosis rate were significantly elevated following mechanical stretching. However, the silencing of Piezo1 in hAVWFs seemed to provide a protection against mechanical stretching-induced injury. It suggests that Piezo1 plays an important role in mechanical stretching-induced fibroblasts apoptosis.

So how does Piezo1 lead to fibroblasts apoptosis upon mechanical stretching? Actin cytoskeletons are composed of proteins that serve a variety of biological functions, including cell contraction, motility, and apoptosis. Various stages of apoptosis are marked by dramatic changes in actin filament organization, which demonstrates the importance of the actin cytoskeleton during apoptosis 26. For example, in the final stages of apoptosis, the actin cytoskeleton is degraded, followed by phagocytosis of apoptotic bodies 27. As evidence mounts that actin mediates and initiates apoptosis signaling, actin plays an important role in morphological hallmarks of apoptosis. In our previous study 14, the disruption of the cellular cytoskeleton increased the apoptosis induced by mechanical stretching. The mechanism of apoptosis induced by cytoskeleton disassembly have been studied by many researches. For example, reports have implicated changes to the dynamics of the actin cytoskeleton in the release of ROS from mitochondria and subsequent cell death 28. At the same time, there are other significant changes in this study that deserve our attention. We found that the disassembly of actin cytoskeleton suppressed the protective effect of Piezo1 silencing from mechanical stretching-induced apoptosis. As mentioned earlier, Piezo1, a mechanosensitive ion channel protein, is activated by mechanical stimuli. Several intracellular processes are triggered by Piezo1 activity, including some that affect the cytoskeleton. For Piezo1, the initial effect of this outside-in mechanical transduction was a large inflow of Ca^2+^, which may even be used to reflect the activation of Piezo1 29. Therefore, piezo1 is an important molecule to convert mechanical signals into chemical signals. During the past years, studies have demonstrated that Ca^2+^ signaling modulates the actin cytoskeleton by acting with actin binding proteins 30. On the other hand, Piezo1 activates the proteins associated with cytoskeletal dynamics, such as Calpain 2, which is central to the cytoskeletal changes 31, 32. Combined with our experimental results, it is clear that Piezo1 is close to the actin cytoskeleton, and that piezo1 regulates the state of its disassembly. In addition, When the cytoskeleton is disrupted, minimal mechanical force can induce high Piezo activity 33. Actin cytoskeleton and Piezo1 may form a positive feedback loop, which synergistically leads to the occurrence of SUI, which will be confirm in our later study. However, in the majority of published literature 34, the intensity of cyclic stretch is much higher than 2666 με (used in the present study). Therefore, only the strength of the mechanical stretching could not explain why the low strain causes cells apoptosis. Indeed, multiple other factors, such as bending, vibration, and shear stress from fluid flow may also affect cell apoptosis of hAVWFs during mechanical stretching loading. Because Piezo1 has distinct sensitivity to these mechanical stimulations 5, 31. Therefore, the special function of Piezo1 plays an important role in the occurrence of SUI induced by mechanical stress.

Collectively, the results of our report illustrate the important role of Piezo1 in the mechanical stretching-induced apoptosis via regulating actin cytoskeleton disassembly in hAVWFs. Moreover, we establish the links among Piezo1, actin cytoskeleton and apoptosis with respect to mechanical stretching-induced SUI. Based on these findings, the strategies to regulate the balance of the Piezo1 expression and actin cytoskeleton disassembly could be a therapeutic target to SUI in the clinical practice.

## Abbreviations

SUI: stress urinary incontinence
hAVWFs: human anterior vaginal wall fibroblasts
MS: mechanical stretching
ICS: International Continence Society
